# Temporal Degradation of Skeletal Muscle Quality on CT as a Prognostic Marker in Septic Shock

**DOI:** 10.3390/diagnostics16020247

**Published:** 2026-01-12

**Authors:** June-sung Kim, Jiyeon Ha, Youn-Jung Kim, Yousun Ko, Kyung Won Kim, Won Young Kim

**Affiliations:** 1 Department of Emergency Medicine, University of Ulsan College of Medicine, Asan Medical Center, Seoul 05505, Republic of Korea; jstyle06@amc.seoul.kr (J.-s.K.); yjkim.em@amc.seoul.kr (Y.-J.K.); 2 Medical Imaging Center, Korea University Medicine, Goyeong Campus, Seoul 02841, Republic of Korea; jiyeon.ha85@kumc.or.kr; 3 Department of Radiology and Research Institute of Radiology, University of Ulsan College of Medicine, Seoul 05505, Republic of Korea; yousun.ko@aim-aicro.com (Y.K.); kyungwon.kim@amc.seoul.kr (K.W.K.); 4 Department of Imaging, Dana-Farber Cancer Institute and Brigham and Women’s Hospital, Harvard Medical School, 450 Brookline Avenue, Boston, MA 02115, USA

**Keywords:** myosteatosis, shock, prognosis, CT imaging, critical care

## Abstract

**Background/Objectives**: Although cross-sectional muscle quality has shown prognostic relevance, the impact of temporal changes in muscle composition in septic shock has not been fully explored. This study aimed to investigate whether deterioration in muscle quality on serial computed tomography (CT) scans is associated with mortality in patients with septic shock. **Methods**: We conducted a retrospective single-center study using a prospectively collected registry of adult patients with septic shock between May 2016 and May 2022. Patients who underwent CT on the day of emergency department (ED) presentation and had a CT performed more than 180 days earlier were included. Muscle quality maps were generated and segmented based on CT attenuation values into normal-attenuation muscle area (NAMA), low-attenuation muscle area (LAMA), and intramuscular adipose tissue area. Differences between the ED and prior CT scans were also calculated. The primary outcome was the 28-day mortality. **Results**: Among the 768 enrolled patients, the 28-day mortality was 18.0%. Both survivors and non-survivors showed a significantly greater increase in LAMA (20.8 vs. 9.8 cm^2^) and a greater decrease in NAMA (−26.0 vs. −18.8 cm^2^). Multivariate analysis identified increased LAMA as an independent risk factor for 28-day mortality (adjusted OR 1.03; 95% CI: 1.01–1.04; *p* < 0.01). **Conclusions**: An increase in LAMA on serial CT scans was associated with higher short-term mortality in patients with septic shock, suggesting that temporal degradation of skeletal muscle quality may serve as a potential prognostic marker.

## 1. Introduction

Although early recognition and adequate management of sepsis have improved overall survival rates, it remains a major cause of mortality worldwide [1]. The Third International Consensus Definitions for Sepsis and Septic Shock suggest the use of the Sequential Organ Failure Assessment (SOFA) score for screening and prognostication. However, the SOFA score primarily reflects current organ dysfunction and may not fully capture the cumulative physiological reserve, metabolic burden, or catabolic status that influence patient outcomes. These gaps highlight the need for complementary biomarkers that better reflect underlying patient frailty and biological response to infection [2]. Additionally, risk stratification for each patient with sepsis is important for personalized management due to heterogeneity. Sarcopenia, defined as a decline in muscle mass and function leading to physical disability, may be useful for risk stratification because patients with critical illnesses tend to experience muscle catabolism and reduced muscle mass [3]. Moreover, sarcopenia is easily measured using abdominopelvic computed tomography (CT), which is usually included in routine clinical practice for infection-focus evaluation in patients with septic shock. Previous research has reported an association between poor prognosis and decreased muscle mass in various disease conditions, including malignancy and postoperative and chronic illness [4,5,6,7]. Our study group has also suggested the usefulness of sarcopenia as a prognostic marker for patients with septic shock; however, sex differences created limitations in prognostic ability, and only male patients showed an association between sarcopenia and short-term mortality [4]. Recent work has also questioned the prognostic accuracy of conventional biochemical or metabolic biomarkers in septic shock. Roukhomovsky-Moretti et al. reported that the strong ion gap, determined using the Stewart approach, was not independently associated with mortality in septic shock [8]. These findings underscore the limitations of traditional biomarkers and highlight the need for complementary imaging-based indicators that reflect both metabolic and inflammatory burden. CT-based muscle metrics may provide such integrated information, bridging metabolic stress and physiological reserve.

Meanwhile, recent guidelines of the European Working Group on Sarcopenia in Older People have suggested that muscle quality is as important as muscle quantity [3]. Myosteatosis, defined as excessive fatty infiltration of the skeletal muscle, may be a parameter of muscle quality and strength [9,10]. It can be measured using CT by discriminating the attenuation of skeletal muscle in Hounsfield Units (HU) on CT scans. Our study group developed a toolkit for automated measurement of muscle quality maps [11]. Fatty infiltration of the muscle occurs during normal aging, but factors associated with critical or chronic illness may accelerate this process. Recent studies have reported the impact of myosteatosis in various patient groups, including those with malignancy, liver cirrhosis, and inflammatory bowel disease [12,13,14,15]. However, its impact and variations in patients with septic shock have not yet been fully explored. Therefore, we hypothesized that the degree of decrease in muscle quality measured by the gap in myosteatosis could be a good prognostic indicator for septic shock. We aimed to investigate the association between changes in muscle quality and short-term mortality in patients with septic shock treated in the emergency department (ED) using protocolized bundle therapy.

## 2. Materials and Methods

### 2.1. Study Design and Population

This retrospective study was based on a prospectively collected registry at a single tertiary university-affiliated hospital in Seoul, South Korea. This registry was designed to enhance internal quality improvement in patients with septic shock and to manage multicenter cohort data of the Korean Shock Society for future analysis. An annual record indicates that approximately 120,000 patients visited the ED between May 2016 and May 2022. All adult patients (age ≥ 18 years) with confirmed septic shock were enrolled in this registry based on the Sepsis-2 and Sepsis-3 guidelines. Persistent hypotension, defined as a systolic blood pressure < 90 mmHg or a mean arterial pressure < 65 mmHg, is considered a refractory shock that requires the use of vasoactive agents [16,17]. Patients excluded from the registry included those who (1) were transferred from or to another hospital, (2) refused management or had a “do-not-resuscitate” order, (3) developed septic shock 6 or more hours after the ED visit, and (4) did not need vasopressors after fluid resuscitation.

The interval between the baseline and ED CT scans was set at ≥180 days to reflect the typical follow-up pattern in our institution. Abdominopelvic CTs are usually repeated at 6–12-month intervals for oncologic or chronic disease surveillance, although in acute or treatment-intensive settings, follow-up may occur at shorter intervals (1–3 months). This criterion was chosen to exclude short-term follow-up scans while ensuring consistency across patients and to capture long-term rather than transient changes in muscle quality.

In our hospital’s ED, a protocolized approach for diagnosis and treatment is followed when a patient with suspected septic shock arrives. This process typically involves obtaining a medical history, conducting a physical examination, and performing laboratory tests such as creatinine and glomerular filtration rate measurements prior to obtaining a CT scan. CT scans are generally performed approximately 4 h after the patient’s arrival in the emergency department.

Following initial resuscitation and diagnostic evaluation in the ED, all patients with confirmed septic shock were subsequently transferred to the intensive care unit (ICU) for ongoing hemodynamic monitoring and management according to the institutional sepsis protocol. The timing of ICU admission depended on bed availability and the patient’s clinical stability after initial stabilization.

### 2.2. Data Collection and Definition of Variables

Demographic data, sources of infection, and various sepsis-related values (SOFA score, acute physiology, chronic health evaluation [APACHE)] II score, and initial lactate level) were extracted from the registry. SOFA and APACHE scores were calculated based on the worst values within 24 h of the ED visit. The primary outcome of this study was the 28-day mortality.

**Figure 1:**
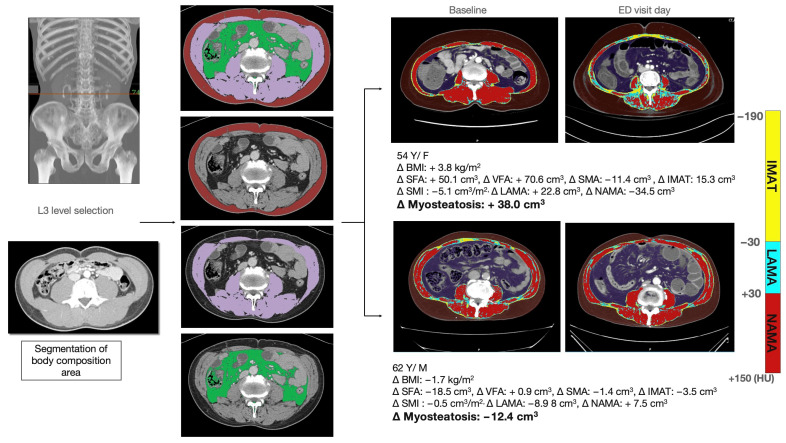
Example of body composition measurements. A muscle-quality map was generated using a web-based toolkit. Body area was divided into three compositions (i.e., subcutaneous fat [SFA], visceral fat [VFA], and skeletal muscle area [SMA]). The skeletal muscle area was further classified into three areas based on the CT density of the area (i.e., normal attenuation muscle area [NAMA], low attenuation muscle area [LAMA], and intermuscular adipose tissue area [IMATA]). An example of a muscle quality decrease is presented in the upper row, and an example of a muscle quality improvement is presented in the lower row.

Body morphometry data were collected from electronic medical records. Enhanced CT in the portal phase was included only in the body composition analysis. Body composition areas, including subcutaneous fat area (SFA), visceral fat area (VFA), and muscle quality maps including normal attenuation muscle area (NAMA), low attenuation muscle area (LAMA), and intramuscular adipose tissue area (IMATA), were measured using APCT (Figure 1). The skeletal muscle area (SMA) was defined as the sum of NAMA and LAMA. All values were consistently measured on a single CT scan at the L3 lower endplate level. Normal attenuation of muscle was defined as a CT number (Hounsfield units [HU]) range between +30 HU and +150 HU, and low attenuation of muscle was defined as a range between −29 HU and +29 HU. Intramuscular adipose tissue was defined as −190 HU to −30 HU. A deep learning-based automatic system (AID-UTM, iAID Inc., Seoul, Republic of Korea) was used for L3 level localization and body composition analysis. The algorithm was trained on a large, annotated dataset of abdominopelvic CT images and validated in multiple independent cohorts for accurate segmentation of skeletal muscle and adipose tissues, demonstrating excellent reproducibility and robustness in clinical practice [11,18]. A board-certified abdominal radiologist confirmed the muscle quality map image generated by the deep learning-based algorithm. The body composition areas for each ER visit day and prior CT scan were measured, and the difference was calculated. The institutional review board of the Asan Medical Center approved this study (IRB number: 2021-0392), and the requirement for informed consent was waived given the retrospective nature of the study.

### 2.3. Statistical Analysis

Demographic and clinical characteristics including underlying illness and site of infection, laboratory data, and areas of body composition are presented as frequencies and percentages for categorical variables. Continuous variables are presented as median and interquartile range (IQR) because of their non-normal distribution. All descriptive statistics were stratified according to the all-cause 28-day mortality. Chi-square or Fisher’s exact test was used for categorical variables. The association between LAMA, LAMA difference, and 28-day mortality was evaluated. Univariate logistic regression tests were used to evaluate potential risk factors for survival, whereas multivariate logistic regression analysis was conducted with variables that indicated statistical significance from the univariate logistic regression. Adjusted odds ratios (OR) and 95% confidence intervals (CI) were calculated. *p*-values was set at *p* < 0.05. SPSS for Mac, version 26 (IBM Corp., Armonk, NY, USA) and R version 3.5.0 (R Foundation for Statistical Computing, Vienna, Austria) were used for the analysis.

## 3. Results

**Figure 2:**
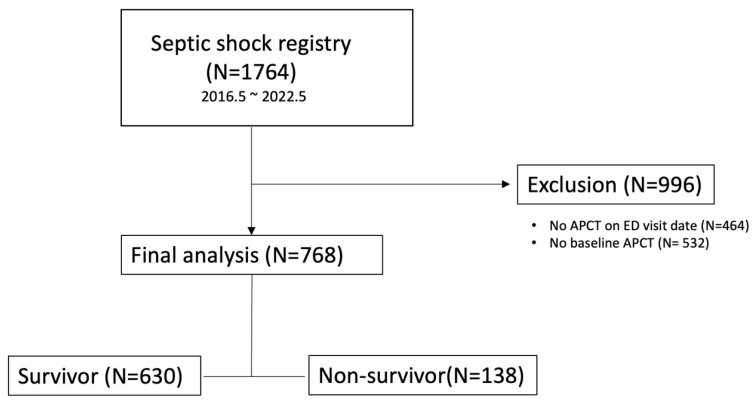
Flowchart of patient enrollment and allocation.

Of the 1764 patients enrolled in the registry, 768 had CTs on both the ED visit day and the previous visit day (Figure 2). Patients without APCT in the ER or previous visit days were excluded (*n* = 996). The 28-day survival rate was 82.0% (*n* = 630).

The baseline characteristics of the study population are summarized in Table 1. The median age of all patients was 66.0 (59.0–74.0), and 61.6% were male. Patients in the non-survival group had more underlying malignancies (63.8% vs. 51.6%, *p* < 0.01), more respiratory tract infections (21.0% vs. 12.1%, *p* < 0.01), fewer hepatobiliary tract infections (35.5% vs. 45.1%, *p* = 0.04), and more bloodstream infections (8.0% vs. 3.3%, *p* = 0.01). Additionally, they had significantly higher lactate levels (5.4 vs. 3.0 mmol/L, *p* < 0.01), SOFA (10.0 vs. 7.0, *p* < 0.01), and APACHE II scores (21.0 vs. 16.0, *p* < 0.01). The interval between CT scans was approximately 325 days, which did not significantly differ between the survival and non-survival groups.

Differences in body morphometric data between the ED visit day and baseline are summarized in Table 2. Larger LAMA (46.4 cm^2^ vs. 55.0 cm^2^, *p* < 0.01) and smaller NAMA (61.2 cm^2^ vs. 48.4 cm^2^, *p* < 0.01) were observed on the day of the emergency department (ED) visit in the non-survivor group. However, there was no significant difference in baseline CT measurements obtained during the prior visit. SFA decreased significantly in the non-survival group on ED visit day (−7.2 cm^2^ vs. −15.7 cm^2^, *p* < 0.01). The non-survivor group had a greater increase in LAMA (9.8 cm^2^ vs. 20.8 cm^2^, *p* < 0.01) and decreased NAMA (−18.8 cm^2^ vs. −26.0 cm^2^, *p* < 0.01) compared with the survivor group.

**Figure 3:**
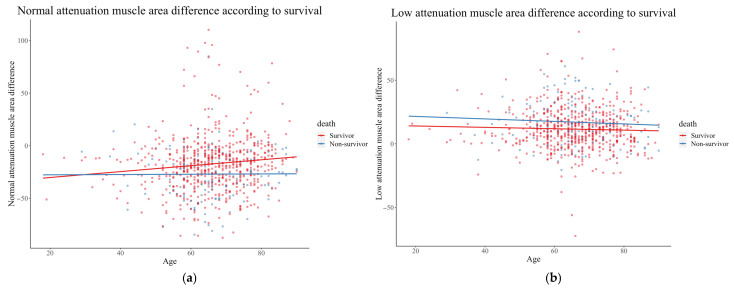
Distribution of normal (**a**) and low attenuated muscle area differences (**b**) according to age in the survivor and non-survivor groups.

Figure 3 shows the differences in low and normal attenuated muscle areas according to age. The difference in NAMA increased in the group age > 30 years. NAMA decreased in both groups but to a lesser extent in the survival group. Additionally, a greater increase in LAMA was observed in the non-survival group than in the survival group, across all age groups.

Logistic regression analysis was conducted to evaluate the association between 28-day mortality and potential influencing factors (Table 3). The presence of underlying malignancy, lung infection, elevated lactate levels, increased SOFA and APACHE scores, decreased SFA, and increased LAMA were associated with 28-day mortality in the univariate analysis. In the multivariate logistic regression analysis, decreased SFA (adjusted OR, 0.99; 95% CI: 0.98–0.99; *p* < 0.01) and increased LAMA (adjusted OR, 1.03; 95% CI: 1.01–1.04; *p* < 0.01) remained independently associated with 28-day mortality.

## 4. Discussion

We observed that a higher baseline-to-sepsis presentation LAMA change in patients with septic shock is significantly associated with short-term mortality. A reduction in muscle quality at the time of ED presentation, compared to a prior visit, was independently associated with short-term mortality in patients with septic shock, regardless of the underlying conditions or infection site.

The association between muscle quality and mortality of various malignancies, such as colorectal, hepatocellular, and pancreaticobiliary cancers, has been reported in previous studies [12,13,19,20,21]. Additionally, the association between short-term mortality from septic shock and low-quality muscle was established in our previous study [22]. The primary focus of this study was the differences in muscle quality and the influence of these differences on mortality in septic shock. We hypothesized that catabolism associated with septic shock may contribute to muscle deterioration, thereby accelerating the fatty infiltration of the muscle. Muscle quality is affected by various conditions, such as diabetes, dyslipidemia, and high-fat diet [23], which reflect the disease condition or nutritional status of a patient.

The difference in low-quality muscle may be a more useful indicator than a single measurement for evaluating disease severity. Most previous studies have used muscle radiodensity as an indicator of muscle quality [8]. The average radiodensity of the muscle area reflects the general condition of the entire muscle and cannot indicate the proportion of healthy and unhealthy muscles. The cut-off value of muscle radiodensity did not differ across the sexes [21]; however, the low-quality muscle area largely depends on the total muscle mass, which differs according to sex.

Myosteatosis is the sum of IMATA and LAMA. The relationship between LAMA and physical functional disability or metabolic alterations has been previously reported. Additionally, LAMA is considered an indicator of poor physical and metabolic prognosis [24,25,26]. IMATA is the visible fat in the muscle, which reflects intramuscular and intermuscular fat and is associated with insulin resistance, lower cancer survival rate, and loss of strength and mobility [27,28,29,30]. Although a significant decrease in LAMA was observed in the non-survivor group, the IMATA change was not significant between the survivor and non-survivor groups. The severity of septic shock may be associated with increased unhealthy muscle, represented as LAMA, rather than apparent fat tissue between the muscle groups.

The exact mechanism underlying the association between decreased muscle quality and mortality has not been fully examined. In the past, muscle quality change was considered an aging process, in which increasing endogenous glucocorticoids could lead to fat deposition [31]. However, recent studies have proposed that pathologic conditions could accelerate the decrease in muscle quality, such as a high-fat diet, sex hormone deficiency, excess stress hormone, and prolonged bed rest [32,33]. Moreover, metabolic dysfunction of skeletal muscles may be correlated with oxidative stress, systemic inflammation, and insulin resistance, which could impede protein composition, muscle synthesis, and organ function [8]. Low muscle quality and short-term mortality in patients with septic shock may not be directly associated, but low-quality muscle areas could be potential biomarkers. Rapid muscle degradation related to sepsis has been well-documented in previous studies, with a reported prevalence of 40% to 70% among sepsis patients. Inflammatory cytokines, including IL-6, TNF-α, IFN-γ, and IL-1, which increase during the early phase of sepsis, are believed to contribute to acute muscle wasting. IL-6 has been shown to directly affect myofibrils, whereas other inflammatory cytokines influence various signaling pathways involved in muscle protein degradation. These cytokines upregulate the expression of muscle-atrophy-related genes. Additionally, factors such as prolonged ventilator use may contribute to respiratory muscle atrophy, including that of the diaphragm [34]. The inflammatory cascade plays a key role in sepsis-induced muscle degradation. Recent therapeutic evidence further supports this concept: Chen et al. demonstrated that cytokine adsorption using HA380 hemoperfusion significantly improved survival in septic shock by attenuating hyperinflammation [35]. This finding reinforces the mechanistic link between inflammation, metabolic catabolism, and skeletal muscle deterioration, suggesting that controlling systemic inflammation could help preserve muscle quality and improve outcomes.

Because the baseline CT was performed more than 180 days before ED presentation, the temporal change in LAMA predominantly reflects a chronic decline in muscle quality, most likely associated with pre-existing frailty, malignancy, or reduced physical activity. Nonetheless, because the exact onset of sepsis is often uncertain, a minor component of acute muscle deterioration during the early phase of sepsis may also be incorporated. Therefore, the LAMA difference should be interpreted mainly as a biomarker of pre-existing physiological reserve rather than as an indicator of acute catabolic change directly caused by the septic episode itself.

This study is the first to demonstrate that temporal degradation of skeletal muscle quality, assessed using serial CT, is associated with short-term mortality in septic shock. By focusing on longitudinal rather than static measurements, our findings highlight the potential of dynamic muscle quality assessment as an adjunctive tool for risk stratification in critical care. However, these results should be interpreted as hypothesis-generating and validated in prospective studies before clinical application.

Our study had several limitations. First, we included only patients with septic shock who underwent CT imaging. As a result, the baseline characteristics of the study population revealed a relatively low incidence of lung infections (13.7%) and a high prevalence of abdominal infections (58.6%). In addition, including only patients who had a prior abdominopelvic CT at least 180 days before sepsis onset may have introduced selection bias toward those with chronic diseases such as malignancies, which could have contributed to the relatively low mortality rate (18.0%). The observed relatively low mortality rate (18.0%) can be attributed to this selection bias. Second, the edematous condition of acutely ill patients may have affected the visceral or subcutaneous fat area by increasing the attenuation of the fat area, causing the algorithm to exclude it from the fat area and risking underestimation. Third, a diagnostic cut-off for muscle quality has not yet been established. An increased low-quality muscle area may predict short-term mortality; however, there is no definite value that defines a significant increase in low-quality muscle area. Fourth, the body morphometric values depend on racial and ethnic factors. Differences between races are widely known [36]. This study was based on a registry in South Korea, the results of which may be difficult to generalize to other populations. Fifth, our registry included patients with septic shock but not sepsis. Although a study involving a population of sepsis patients could provide more insight into the characteristics of this disease entity, it posed challenges to screen all suspected sepsis patients in the ED, particularly because of the relatively high rate of false positives in diagnosing infection [37]. Sixth, although we performed multivariate logistic regression accounting for suspected confounders in the registry, there may still be hidden confounders that could affect muscle quality, such as baseline activities of daily living, nutritional status, and socioeconomic status. Finally, the effect size of LAMA on mortality was modest (aOR, 1.03). Although the magnitude appears small, this likely reflects differences in measurement scale rather than a lack of biological or clinical relevance. The use of a more precise measurement unit in this study may have attenuated the apparent effect size because of potential variability or measurement noise. Nonetheless, presenting the findings in the current units facilitates a more nuanced interpretation of the study’s primary question, although further studies are warranted to establish clinically meaningful thresholds for muscle quality change.

## 5. Conclusions

In summary, a greater increase in LAMA between the baseline and sepsis-presentation CT scans was independently associated with short-term mortality in patients with septic shock. The temporal trend of muscle quality may serve as a potential indicator of disease severity and nutritional status in this population. Although our findings suggest that mitigating muscle deterioration could improve outcomes, further prospective research is warranted to confirm its clinical and therapeutic implications.

## Figures and Tables

**Table 1:** Baseline characteristics of patients with septic shock according to 28-day mortality.

Characteristics	Total	Survivor	Non-Survivor	*p*
	(*n* = 768)	(*n* = 630)	(*n* = 138)	
Age, year	66.0 (59.0–74.0)	67.0 (59.0–74.0)	65.0 (58.0–71.0)	0.48
Male	473 (61.6)	386 (61.3)	87 (63.0)	0.70
Past illness				
HTN	231 (30.1)	186 (29.5)	45 (32.6)	0.47
DM	200 (26.0)	166 (26.3)	34 (24.6)	0.68
CAD	73 (9.5)	59 (9.4)	14 (10.1)	0.78
Chronic pulmonary disease	50 (6.5)	38 (6.0)	12 (8.7)	0.25
Malignancy	413 (53.8)	325 (51.6)	88 (63.8)	<0.01
Hematologic disorder	46 (6.0)	35 (5.6)	11 (8.0)	0.28
CKD	56 (7.3)	47 (7.5)	9 (6.5)	0.70
LC	142 (18.5)	112 (17.8)	30 (21.7)	0.28
Site of infection				
Unknown	74 (9.6)	64 (10.2)	10 (7.2)	0.29
Lung	105 (13.7)	76 (12.1)	29 (21.0)	<0.01
Urinary tract	111 (14.5)	92 (14.6)	19 (13.8)	0.80
Gastrointestinal	117 (15.2)	89 (14.1)	28 (20.3)	0.07
Hepato-biliary-pancreas	333 (43.4)	284 (45.1)	49 (35.5)	0.04
Bloodstream	32 (4.2)	21 (3.3)	11 (8.0)	0.01
Lactate level, mmol/L	3.3 (1.8–5.6)	3.0 (1.7–5.2)	5.4 (2.9–8.5)	<0.01
SOFA score	7.0 (5.0–10.0)	7.0 (5.0–9.0)	10.0 (6.0–12.0)	<0.01
APACHE score	17.0 (13.0–22.0)	16.0 (12.0–21.0)	21.0 (17.0–27.5)	<0.01
CT interval *	325 (182–377)	321 (174–380)	330 (182–369)	0.65

Data are presented as *n* (%) or median (interquartile range). * The interval between baseline CT and ED visit day was calculated and presented as days. Abbreviations: HTN, hypertension; DM, diabetes mellitus; CAD, coronary artery disease; CKD, chronic kidney disease; LC, liver cirrhosis; SOFA, sequential organ failure assessment; APACHE, Acute Physiology and Chronic Health Evaluation.

**Table 2:** Body composition of the study population.

Body Composition	Total	Survivors	Non-Survivors	*p*
	(*n* = 768)	(*n* = 630)	(*n* = 138)	
SFA, cm^2^				
ED visit day	96.2 (55.9–145.0)	92.9 (59.8–147.3)	92.2 (37.3–146.9)	0.34
Baseline *	113.3 (75.5–159.2)	101.5 (73.5–155.0)	103.3 (65.6–185.8)	0.31
Difference **	−13.2 (−39.0–9.2)	−7.2 (−33.6–14.6)	−15.7 (32.6–9.3)	<0.01
VFA, cm^2^				
ED visit day	88.6 (51.7–139.3)	92.3 (52.0–140.6)	97.0 (67.9–142.7)	0.79
Baseline *	94.9 (53.7–144.7)	98.3 (57.0–150.3)	108.1 (59.5–126.0)	0.43
Difference **	−4.2 (−28.0–18.2)	−7.9 (−30.0–17.6)	−8.1 (−27.3–29.1)	0.84
SMA, cm^2^				
ED visit day	105.3 (90.3–123.9)	105.9 (93.5–128.5)	109.6 (87.4–117.1)	0.44
Baseline *	113.4 (96.5–138.0)	115.4 (99.9–139.1)	113.1 (97.3–143.3)	0.82
Difference **	−8.2 (−20.1–0.0)	−7.9 (−19.1–0.7)	−11.4 (−21.3–3.3)	0.30
LAMA, cm^2^				
ED visit day	46.9 (35.4–59.2)	46.4 (34.2–59.0)	55.0 (48.4–67.0)	<0.01
Baseline *	34.2 (26.7–44.5)	37.3 (27.9–54.6)	35.3 (27.6–47.2)	0.16
Difference **	11.1 (2.3–20.4)	9.8 (1.7–18.8)	20.8 (5.5–29.3)	<0.01
NAMA, cm^2^				
ED visit day	56.8 (39.6–77.3)	61.2 (41.7–78.6)	48.4 (31.0–60.3)	<0.01
Baseline *	78.3 (59.7–101.9)	80.8 (62.1–100.8)	72.2 (54.1–108.3)	0.76
Difference **	−19.7 (−35.2–−7.6)	−18.8 (−31.8–−5.3)	−26.0 (−45.9–−7.2)	<0.01
IMATA, cm^2^				
ED visit day	14.9 (8.8–22.1)	14.9 (8.7–20.5)	14.3 (9.3–22.3)	0.77
Baseline *	14.0 (9.1–19.9)	13.9 (8.8–19.9)	12.1 (8.7–18.3)	0.16
Difference **	0.6 (−3.3–5.1)	0.5 (−3.1–4.4)	3.3 (−1.6–8.2)	0.84

Data are presented as median (interquartile range). * Baseline was extracted from previous computed tomography for routine surveillance without severe illness for more than 180 days before the ED visit. ** Difference (Δ) values were calculated as the measurement obtained at the ED presentation minus the corresponding baseline value. Abbreviations: SFA, subcutaneous fat area; ED, Emergency Department; VFA, visceral fat area; SMA, skeletal muscle area; LAMA, low attenuation muscle area; NAMA, normal attenuation muscle area; IMATA, intramuscular adipose tissue area.

**Table 3:** Multivariate analysis of patients with septic shock for the association with 28-day mortality.

Variables	Univariate Analysis			Multivariate Analysis		
	OR	95% CI	*p*-Value	aOR	95% CI	*p*-Value
Underlying malignancy	2.19	1.36–3.55	<0.01	2.06	1.29–3.30	<0.01
Site of infection						
Lung	2.10	1.13–3.90	0.02	2.15	1.22–3.79	<0.01
Gastrointestinal	1.27	0.68–2.37	0.45			
Hepato-biliary-pancreas	0.86	0.50–1.47	0.58			
Bloodstream	2.36	0.94–5.90	0.07	2.39	0.97–5.90	0.06
Lactate level	1.14	1.07–1.22	<0.01	1.14	1.07–1.22	<0.01
SOFA score	1.12	1.03–1.20	<0.01	1.12	1.04–1.20	<0.01
APACHE score	1.03	1.01–1.08	0.02	1.04	1.01–1.08	0.01
Body composition area						
SFA difference	0.99	0.98–0.99	<0.01	0.99	0.98–1.00	0.06
ED visit day LAMA	1.01	1.00–1.03	0.16			
LAMA difference	1.02	1.00–1.04	0.07	1.03	1.01–1.04	<0.01
ED visit day NAMA	0.99	0.98–0.99	<0.01	0.99	0.98–1.00	0.06
NAMA difference	0.98	0.98–1.01	0.08	0.99	0.97–1.03	0.09

OR, odds ratio; CI, confidence interval; SOFA, sequential organ failure assessment; APACHE, Acute Physiology and Chronic Health Evaluation; SFA, subcutaneous fat area; ED, emergency department; LAMA, low attenuated muscle area.

## Data Availability

Data of this study is unavailable due to privacy or ethical restrictions.

## Abbreviations

The following abbreviations are used in this manuscript:

SOFA	Sequential organ failure assessment
APCT	Abdominopelvic computed tomography
HU	Hounsfield Units
ED	Emergency Department
APACHE	Acute Physiology and Chronic Health Evaluation
SFA	Subcutaneous fat area
VFA	Visceral fat area
NAMA	Normal attenuation muscle area
LAMA	Low attenuation muscle area
IMATA	Intramuscular adipose tissue area
IQR	Interquartile range
OR	Odds ratio
CI	Confidence interval
